# Antifungal Activity of Methyl 2-Methylbutyrate Against *Botrytis cinerea* and the Induced Physiological, Transcriptional, and Metabolic Responses

**DOI:** 10.3390/ijms27198589

**Published:** 2026-09-25

**Authors:** Li-Ming Dai, Li-Li He, Si-Min Li, Yi-Xian Liu, Yu-Ping Shi, Hai-Peng Su, Zhi-Ying Cai

**Affiliations:** Tropical Crop Diseases Research Group, Research Center of Plant Protection, Yunnan Key Laboratory of Sustainable Utilization Research on Rubber Tree, Yunnan Institute of Tropical Crops, Jinghong 666100, China; limingdai@126.com (L.-M.D.);

**Keywords:** methyl 2-methylbutyrate, *Botrytis cinerea*, microbial volatile organic compounds, transcriptomics, metabolomics, redox response

## Abstract

Gray mold, caused by the necrotrophic fungus *Botrytis cinerea*, is a devastating disease responsible for substantial global crop losses. The escalating threat of fungicide resistance and environmental concerns necessitates the development of eco-compatible control alternatives. In this study, we investigated the antifungal efficacy of methyl 2-methylbutyrate (M2M), a microbial volatile organic compound (mVOC), against *B. cinerea*, and elucidated the underlying physiological, transcriptomic, and metabolomic responses. Our results demonstrated that M2M significantly inhibited mycelial growth in a dose-dependent manner, with an EC_50_ value of 3.49 μL/plate. Scanning electron microscopy revealed severe ultrastructural damage in M2M-treated hyphae, characterized by localized swelling, shriveling, and irregular bending. Physiological assays indicated that M2M significantly upregulated the activities of antioxidant enzymes, including catalase (CAT), peroxidase (POD), and superoxide dismutase (SOD), while significantly reducing malondialdehyde (MDA) content, suggesting that M2M triggers an oxidative challenge to which *B. cinerea* responds by activating its antioxidant defense system, thereby suppressing lipid peroxidation and the activities of key metabolic enzymes, such as succinate dehydrogenase (SDH), glucose-6-phosphate dehydrogenase (G6PDH), and lactate dehydrogenase (LDH). These findings suggest that M2M treatment is associated with perturbations in cellular redox homeostasis and central carbon metabolism in *B. cinerea*. Transcriptomic profiling identified 3664 differentially expressed genes (DEGs), predominantly enriched in carbon metabolism, glyoxylate and dicarboxylate metabolism, redox pathways, and ribosomal biogenesis. Notably, M2M exposure significantly perturbed the transcriptional levels of ROS-scavenging genes (e.g., *CAT*, *POD*, *GPX*, *PRX*, and *GST*) and various transcription factors. Untargeted metabolomics revealed 1041 differentially accumulated metabolites (DAMs), with lipids and lipid-like molecules representing the most significantly altered class. Integrated multi-omics analysis revealed that M2M treatment was associated with coordinated changes in lipid metabolism, central carbon metabolism, and redox balance. This study provides mechanistic insights into the antifungal action of M2M and suggests that M2M warrants further investigation as a potential candidate for postharvest gray mold management.

## 1. Introduction

Gray mold, caused by the necrotrophic fungus *Botrytis cinerea* (Pers. Fr., teleomorph: *Botryotinia fuckeliana*), is one of the most devastating fungal diseases affecting global agricultural production. This pathogen has a remarkably broad host range, infecting over 1400 plant species across nearly 600 genera, including numerous economically important crops such as tomato, strawberry, grape, and cucumber, leading to substantial yield losses and economic burdens annually [1]. The high genetic plasticity, rapid reproductive cycle, and adaptability of *B. cinerea* facilitate the rapid evolution of fungicide resistance [2]. Coupled with growing concerns over pesticide residues and environmental contamination, there is an urgent imperative to develop eco-friendly and effective alternatives. Biological control, particularly the utilization of antagonistic microorganisms and their bioactive metabolites, represents a sustainable and promising strategy to mitigate gray mold [3].

In recent years, microbial volatile organic compounds (mVOCs) have emerged as compelling candidates for sustainable disease management, owing to their high diffusibility, contact-independent bioactivity, and minimal environmental persistence [4,5]. Accumulating evidence indicates that mVOCs suppress phytopathogens by disrupting plasma membrane integrity, inducing oxidative bursts, interfering with energy metabolism, and reprogramming gene expression profiles [6]. Against *B. cinerea*, several mVOCs from antagonistic microbes have exhibited significant antifungal effects. For instance, VOCs from *Aureobasidium pullulans* were shown to inhibit pathogen growth by inducing ROS accumulation, promoting lipid peroxidation, and causing cellular content leakage in *B. cinerea* [7]. Similarly, VOCs from *Streptomyces globisporus* JK-1 and *A. pullulans* strains L1 and L8 effectively reduced the incidence and spread of gray mold on tomato and apple fruits [8,9]. Notable examples include VOCs from *Pseudomonas chlororaphis* [10] and *Bacillus siamensis* [11], which have been shown to suppress mycelial growth, spore germination, and disease development through multi-target mechanisms. These findings indicate that mVOCs can modulate the cellular structure and metabolic networks of pathogenic fungi through multiple pathways and at various levels.

Methyl 2-methylbutyrate (M2M), a naturally occurring ester prevalent in the aroma profiles of apples, strawberries, and pineapples, is generally recognized as safe (GRAS) and approved as a food flavoring agent under the Chinese National Standard GB 2760-2024 [12]. This regulatory status underscores its high biosafety profile and commercial viability for agricultural applications. Our research group previously isolated a strain of *Pseudomonas aeruginosa* BACYitc8-7 from soil and identified M2M as one of the primary active components in its mVOC profile. Previous studies have reported that M2M exhibits significant inhibitory effects against several fungi. Lyu et al. [13] found that M2M could inhibit conidial germination in *Aspergillus flavus*. M2M has been reported to exhibit antifungal activity against *A. flavus* [14], *Monilinia fructicola* [15], and *Penicillium digitatum* [16], with mechanisms involving cell wall/membrane disruption, oxidative damage, and interference with energy metabolism. Beyond these individual reports, our research group has recently demonstrated the broad-spectrum antifungal potential of M2M against a diverse panel of economically important phytopathogens through a patent application [17]. Notably, M2M exhibited potent inhibitory activity against *Colletotrichum* spp., *Alternaria* spp., and other fungal pathogens. These findings highlight the broad-spectrum antifungal activity of M2M and prompted us to investigate its efficacy and underlying mechanisms against *B. cinerea*, a pathogen of global agricultural significance. Importantly, the acid form of M2M, 2-methylbutyric acid (2-MBA), has been shown to inhibit *B. cinerea* growth by inducing oxidative damage, interfering with the TCA cycle and energy metabolism, and downregulating the expression of pathogenicity-related genes. Furthermore, 2-MBA was able to induce disease resistance in cherry tomatoes, effectively controlling postharvest gray mold development [18]. Despite these advances, a systematic investigation into the antifungal efficacy and multi-omics mechanisms of M2M against *B. cinerea* remains conspicuously absent.

Therefore, this study aimed to systematically investigate the phenotypic, physiological, transcriptional, and metabolic responses of *B. cinerea* to M2M treatment through phenotype analysis, microscopic observation, physiological index measurements, and integrated transcriptomic and untargeted metabolomic analyses. We sought to identify the biological processes associated with the inhibition of *B. cinerea* by M2M, thereby providing a mechanistic framework for its potential application as a green fumigant for postharvest gray mold management.

## 2. Results

### 2.1. M2M Inhibits Mycelial Growth and Induces Structural Abnormalities in B. cinerea

To evaluate the antifungal activity of methyl 2-methylbutyrate (M2M) against *Botrytis cinerea*, the double-dish inverted culture method was used to assess the effect of different M2M concentrations on mycelial growth. Following a 5-day incubation, M2M treatment markedly suppressed the colony expansion of *B. cinerea* relative to the untreated control. The inhibitory effect exhibited a robust dose-dependent relationship, with increasing concentrations of M2M resulting in progressively severe growth restriction (Figure 1A,B). The inhibition rates at M2M concentrations of 3, 5, 7, 10, and 15 μL/plate were 50.99%, 64.32%, 72.81%, 97.89%, and 100%, respectively. The calculated half-maximal effective concentration (EC_50_) for inhibiting mycelial growth was 3.49 μL/plate, indicating a strong inhibitory activity of M2M against *B. cinerea* (Table 1).

Scanning electron microscopy (SEM) revealed striking ultrastructural alterations in M2M-treated hyphae (Figure 1C). While control hyphae exhibited smooth surfaces, uniform diameters, and regular linear extension, treated hyphae displayed pronounced morphological aberrations, including diameter heterogeneity, localized swelling, cytoplasmic shrinkage (shriveling), and erratic bending. These results indicate that M2M not only effectively inhibits mycelial growth but also disrupts the normal mycelial morphology, suggesting that M2M treatment may compromise the structural integrity of the fungal mycelium, thereby impairing its normal growth.

### 2.2. M2M Affects Redox Status and Key Metabolic Enzyme Activities in B. cinerea

To explore the physiological effects of M2M treatment on *B. cinerea* cells, the changes in redox-related indicators and the activities of key central metabolic enzymes were measured. For physiological analyses, M2M was applied at 3 μL/plate (approximately the EC_50_). M2M treatment induced significant perturbations in the physiological and biochemical profiles of *B. cinerea* (Figure 2; Table 2). Regarding redox homeostasis, M2M exposure significantly elevated the activities of the antioxidant enzymes CAT (+21.1%, *p* = 0.024), SOD (+87.4%, *p* = 0.008), and POD (+63.3%, *p* = 0.001) relative to the control, while the MDA content was reduced by 59.6% (*p* < 0.001), indicating that the M2M-induced activation of the antioxidant defense system (as reflected by elevated SOD, CAT, and POD activities) effectively suppressed lipid peroxidation and limited oxidative membrane damage. Furthermore, M2M treatment significantly altered the activities of central metabolic enzymes: SDH and G6PDH activities increased by 94.1% (*p* = 4.11 × 10^−6^) and 52.2% (*p* = 0.0002), respectively, while LDH activity decreased by 57.4% (*p* < 0.0003) (Table 2). These results suggest that M2M treatment not only affects the antioxidant defense system but is also accompanied by changes in the activities of enzymes related to energy and carbohydrate metabolism. The raw data for all physiological parameters, including individual replicate values, are provided in Supplementary Table S1.

The effect sizes (Cohen’s d) for all parameters ranged from 10.02 to 75.60, all well above the threshold for large effects (*d* > 0.8), and the 95% confidence intervals for all mean differences did not contain zero (Table 2), confirming that the observed differences are both statistically significant and biologically meaningful.

### 2.3. Transcriptomic Analysis Reveals Global Gene Expression Changes in B. cinerea After M2M Treatment

For transcriptomic analysis, M2M was applied at 3 μL/plate (approximately the EC_50_). To dissect the molecular responses induced by M2M treatment, transcriptome sequencing analysis was performed on mycelial samples collected after 5 days of incubation from the M2M-treated group and the control group. After quality control, a total of 284,087,540 clean reads were obtained from the six samples, with high Q20 and Q30 scores for all samples (Supplementary Table S2), indicating reliable sequencing data quality. The transcriptome data have been deposited in the NCBI SRA database (BioProject accession: PRJNA1358801).

Principal component analysis (PCA) showed clear separation between the M2M-treated and control samples in the principal component space, with PC1 and PC2 explaining 42.95% and 22.76% of the total variance, respectively (Figure 3A). This indicates that M2M treatment significantly altered the overall transcriptional profile of *B. cinerea*. Based on the criteria of |log_2_FC| > 1.5 and unadjusted *p* < 0.05, a total of 3664 DEGs were identified, including 1846 upregulated and 1818 downregulated genes (Figure 3B).

KEGG enrichment analysis revealed that upregulated DEGs were mainly enriched in pathways related to carbon metabolism, such as pentose and glucuronate interconversions, glyoxylate and dicarboxylate metabolism, and pyruvate metabolism (Figure 3C), suggesting that M2M treatment affected central metabolic processes. Downregulated DEGs were mainly enriched in pathways such as eukaryotic ribosome biogenesis, RNA polymerase, and purine metabolism (Figure 3D), indicating that M2M treatment may affect RNA processing and protein synthesis-related processes. GO enrichment analysis further showed that upregulated DEGs were primarily involved in functions such as oxidoreductase activity, NAD binding, membrane components, and transmembrane transport (Figure 3E), while downregulated DEGs were mainly associated with ribosome biogenesis, rRNA processing, and RNA metabolic processes (Figure 3F).

In summary, transcriptomic analysis indicated that M2M treatment induced widespread changes in gene expression in *B. cinerea*, mainly involving central metabolism, redox processes, and RNA metabolism-related functions.

### 2.4. M2M Treatment Alters the Expression Pattern of ROS Metabolism-Related Genes in B. cinerea

To elucidate the molecular basis of the redox response induced by M2M treatment, the expression patterns of genes related to ROS metabolism and antioxidant defense were analyzed based on the DEG results, including members of the *CAT*, *POD*, *GPX*, *GST*, and *PRX* gene families (Figure 4A). The results showed that M2M treatment significantly affected the expression pattern of ROS metabolism-related genes in *B. cinerea*. Among these, Bcin05g04580 (*CAT*), Bcin06g04520 (*CAT*), Bcin11g00390 (*GST*), Bcin11g03930 (*POD*), and Bcin10g02270 (*GST*) showed an upregulated trend in the M2M treatment group (Y), while some members of the *CAT*, *PRX*, *GPX*, *POD*, and *GST* families were downregulated after treatment (Figure 4A). These results indicate that M2M stress can induce differential expression responses of ROS metabolism-related genes in *B. cinerea*.

Spearman correlation analysis was performed between the expression levels of ROS-related genes and the activities of SOD, CAT, POD, and MDA content (Figure 4B). The results showed significant correlations between several ROS-related genes and the redox-related physiological indicators. Further scatter plot analysis of representative significant correlation pairs (Figure 4C) showed that *Bcin10g02270* (GST) expression was significantly positively correlated with SOD activity (*r* = 0.886, *p* = 0.0333), *Bcin06g04520* (CAT) expression was significantly positively correlated with POD activity (*r* = 0.943, *p* = 0.0167), while *Bcin11g03930* (POD) expression was significantly negatively correlated with MDA content (*r* = −0.886, *p* = 0.0333). Additionally, *Bcin08g00300* (GST) expression showed a negative correlation with CAT activity (*r* = −0.886, *p* = 0.0333).

### 2.5. M2M Treatment Affects the Expression Pattern of Transcription Factors in B. cinerea

To further explore the potential transcriptional regulatory factors involved in the M2M response, transcription factor (TF) families were identified among the DEGs. A total of 43 differentially expressed transcription factors were identified (Figure 4D), belonging to families such as CP2, MYB, zf-C2H2, HMG, Homeobox, bHLH, ZBTB, zf-GATA, zf-LITAF-like, and NDT80/PhoG. Expression pattern analysis showed that different TF family members responded differentially to M2M treatment. Most ZBTB family members showed an induced expression trend, while all zf-C2H2 family members showed decreased expression levels after M2M treatment (Figure 4D). These differentially expressed transcription factors may be involved in the regulation of gene expression during the response of *B. cinerea* to M2M stress, providing candidate targets for further dissection of the transcriptional regulatory network underlying the antifungal action of M2M.

### 2.6. M2M Treatment Alters the Global Metabolic Profile of B. cinerea

For metabolomic analysis, M2M was applied at 3 μL/plate (approximately the EC_50_). To analyze the impact of M2M treatment on the overall metabolic profile of *B. cinerea*, an untargeted metabolomics approach was used to analyze samples from the M2M-treated and control groups. Based on chemical structural characteristics, the identified metabolites were classified into categories including lipids and lipid-like molecules, organic acids and derivatives, organoheterocyclic compounds, and benzenoids, with lipids and lipid-like molecules accounting for a high proportion (Figure 5A).

PCA showed clear separation between the M2M-treated and control samples in the principal component space (Figure 5B), indicating that M2M treatment caused a significant shift in the global metabolic profile of *B. cinerea*. OPLS-DA further demonstrated a clear separation between the two groups (Figure 5C). The model parameters were as follows: R^2^X (cum) = 0.682, R^2^Y (cum) = 1.000, and Q^2^ (cum) = 0.981, indicating strong explanatory power and excellent predictive ability of the model. A 200-fold permutation test (Figure 5D) yielded R^2^ and Q^2^ intercepts of 0.900 and −0.426, respectively. The negative Q^2^ intercept (<0) from the permutation test confirms that the original model was not overfitted [19,20].

### 2.7. M2M Treatment Affects the Accumulation of DAMs and Metabolic Pathway Changes in B. cinerea

Based on the untargeted metabolomics data, differentially accumulated metabolites (DAMs) between the M2M-treated group (M2M) and the control group (CK) were screened. Using the criteria of VIP > 1, *p* < 0.05, and |log_2_FC| > 1, a total of 1041 DAMs were identified, of which 654 were upregulated and 387 were downregulated (Figure 6A), indicating that M2M treatment significantly affected the metabolite accumulation pattern in *B. cinerea*. Classification of DAMs showed that they were predominantly distributed among lipids and lipid-like molecules, organoheterocyclic compounds, organic acids and derivatives, and benzenoids (Figure 6B). Notably, the proportion of lipids and lipid-like molecules increased from 54.68% in the control group to 72.96% in the M2M-treated group, becoming the major class of DAMs, suggesting that M2M treatment had a pronounced effect on lipid-related metabolism.

KEGG pathway enrichment analysis showed that DAMs were mainly enriched in carbon metabolism and glyoxylate and dicarboxylate metabolism pathways (Figure 6C). Additionally, alanine, aspartate, and glutamate metabolism also showed an enrichment trend. These results suggest that M2M treatment may affect central carbon metabolism and related energy metabolic processes in *B. cinerea*. Further analysis of representative metabolites in the aforementioned pathways revealed that M2M treatment significantly affected the accumulation levels of key metabolites, including D-gluconic acid, L-glutamic acid, L-glutamine, isocitrate, L-aspartic acid, and citric acid (Figure 6D), indicating that M2M treatment was associated with changes in central carbon metabolism and amino acid metabolism in *B. cinerea*.

### 2.8. Integrated Transcriptomic and Metabolomic Analysis Reveals M2M-Responsive Metabolic Pathways

To explore the associations between gene expression changes and metabolite accumulation after M2M treatment, integrative analysis of transcriptomic and metabolomic data was performed, and the pathways co-enriched by DEGs and DAMs were identified (Figure 7A). The results showed that M2M treatment mainly affected carbon metabolism, lipid metabolism, and redox-related metabolic processes. Among these, the carbon metabolism pathway contained 6 DAMs and 19 DEGs. Additionally, glyoxylate and dicarboxylate metabolism, glycerophospholipid metabolism, glutathione metabolism, and fatty acid-related metabolic pathways also showed obvious co-variation in genes and metabolites (Figure 7A). These results indicated that M2M treatment induced synergistic responses in multiple key metabolic networks in *B. cinerea*.

Spearman correlation analysis was further performed between DEGs and DAMs in key metabolic pathways. A total of 702 Spearman correlation pairs were tested, and a gene-metabolite correlation network was constructed based on |*r*| > 0.75 and *p* < 0.05 using unadjusted *p*-values (Figure 7B). The results revealed significant correlations between various central metabolites, lipid-related metabolites, and redox-related metabolites with functional genes. In lipid metabolism, palmitic acid was significantly positively correlated with the fatty acid synthase-related gene *FAS2*, while it showed a significant negative correlation with *fabD* (*p* < 0.05). Furthermore, the phospholipid metabolism-related gene *EPT1* was significantly positively correlated with various glycerophospholipid metabolites, such as PC and LysoPC (*p* < 0.05), indicating that changes in lipid metabolite accumulation were closely associated with the expression of genes involved in fatty acid synthesis and phospholipid metabolism. In central carbon metabolism, key metabolites including citric acid, isocitrate, L-glutamic acid, and D-gluconic acid showed significant correlations with multiple metabolism-related genes. For example, citric acid and L-glutamic acid were positively correlated with *PC* and *EPT1*, while negatively correlated with *GGT* and *GST*. Isocitrate was positively correlated with *fabG* but negatively correlated with *ME2* and *CDS*. Additionally, redox-related genes also showed significant associations with some metabolites. For instance, the *CAT* gene (*Bcin05g04580*) was significantly correlated with multiple metabolites. Glutathione showed a significant negative correlation with the *GST* gene (*Bcin09g00820*) (*r* = −0.941, *p* = 0.0051). These results indicate that M2M treatment affected the lipid metabolism, central carbon metabolism, and redox-related metabolic networks in *B. cinerea*.

### 2.9. qRT-PCR Validation of Transcriptome Data Reliability

The transcriptomic analysis revealed that M2M treatment significantly altered the expression patterns of multiple functional gene categories in *B. cinerea* (Figure 8A). To validate the reliability of the RNA-seq data, 10 DEGs were selected for qRT-PCR validation. The results showed that the qRT-PCR results exhibited consistent expression trends with the RNA-seq data (Figure 8B), confirming the high reliability of the transcriptome data.

Further analysis revealed that M2M treatment affected the expression of multiple genes associated with pathogen physiological functions. Among these, the pectin degradation-related genes *Bcpg4* and *Bcpg6*, and the cell wall degradation-related genes *Bcpme2* and *Bclip2*, showed upregulated expression trends in the treatment group. Additionally, the NADPH oxidase-related gene *BcnoxD* also showed significant expression changes. In contrast, several genes involved in carbon metabolism and redox regulation, including *Bclga1*, *Bcgar2*, *Bcbas1*, *BcnoxA*, and *Bclip1*, were downregulated after M2M treatment. These results further validated the transcriptomic findings and revealed that M2M treatment could induce the remodeling of gene expression related to cell wall processes, carbon metabolism, and redox regulation in *B. cinerea*.

## 3. Discussion

*Botrytis cinerea*, as a typical necrotrophic plant pathogenic fungus, possesses a broad host range and strong environmental adaptability, making it a significant disease agent in agriculture [21,22]. However, the long-term reliance on chemical fungicides for gray mold management readily leads to the development of pathogen resistance, limiting the sustainable application of traditional control strategies [23,24]. Therefore, the development of natural compounds with antifungal activity and environmental compatibility as alternatives to chemical fungicides has gained widespread attention. In recent years, microbial volatile organic compounds (mVOCs) have attracted increasing interest in the field of green plant disease management due to their strong volatility, high diffusibility, low residue, and multi-target antifungal characteristics [25,26]. In this study, we found that methyl 2-methylbutyrate (M2M) significantly inhibited the mycelial growth of *B. cinerea* and induced structural abnormalities, such as swelling, shriveling, and irregular bending. This suggests that M2M can directly affect the structural stability of fungal hyphae. Previous studies have shown that various volatile compounds can inhibit pathogen growth by disrupting cell structural integrity. For instance, (E)-2-hexenal treatment induced mycelial morphological abnormalities in *B. cinerea*, accompanied by membrane dysfunction, altered redox metabolism, and changes in various physiological processes [27]. Similarly, isooctanol treatment caused mycelial distortion and intracellular ROS accumulation in *B. cinerea* [28]. Furthermore, VOCs from *Chromobacterium vaccinii* induced structural abnormalities, including mycelial swelling, vacuole accumulation, and cell wall alterations, in various plant pathogenic fungi [29]. Therefore, the M2M-induced mycelial structural abnormalities observed in our study are consistent with previously reported mVOC-mediated structural damage in fungi, suggesting that disruption of mycelial structure may be an important early response in the antifungal action of M2M.

Beyond structural damage, our study further revealed that M2M significantly altered the redox status of *B. cinerea*. The increased activities of CAT, POD, and SOD, along with the decreased MDA content after M2M treatment, suggest that *B. cinerea* may have initiated an adaptive antioxidant defense response under M2M stress. Plant pathogenic fungi typically maintain cellular redox homeostasis by regulating SOD, CAT, peroxidase, and glutathione-related enzyme systems to adapt to exogenous oxidative stress [30]. Thus, the M2M-induced increases in CAT, POD, and SOD activities in our study may reflect an adaptive response of *B. cinerea* to oxidative pressure, while the reduction in MDA content, together with the elevated activities of SOD, CAT, and POD, indicates that M2M-induced oxidative challenge was effectively counteracted by the antioxidant defense system, thereby limiting membrane lipid peroxidation MDA is a widely used biomarker of lipid peroxidation, and its accumulation typically reflects oxidative membrane damage [31,32]. In our study, the concurrent increase in antioxidant enzyme activities (SOD, CAT, and POD) was associated with reduced MDA levels. This pattern is consistent with the interpretation that M2M exposure may have imposed an oxidative challenge, to which *B. cinerea* responded by activating its antioxidant defense system. The enhanced ROS-scavenging capacity of SOD and CAT, two of the most important antioxidant enzymes in fungi [33], may have subsequently limited the extent of lipid peroxidation, resulting in reduced MDA accumulation. Similar observations have been reported in other fungal systems: a significant decrease in MDA content was associated with alleviation of lipid peroxidation when antioxidant defenses were engaged [34], and essential oil compounds that enhanced CAT, SOD, and POD activities also led to reduced MDA levels in *B. cinerea*-infected tissues [35]. Thus, the decreased MDA content does not indicate an absence of oxidative stress, but rather reflects the effective engagement of cellular antioxidant defenses that appear to have contained oxidative damage. This interpretation is consistent with the transcriptional upregulation of multiple ROS-scavenging genes observed in our transcriptomic data.

A particularly notable finding was the significant upregulation of SDH activity following M2M exposure, which contrasts with the typical suppression of mitochondrial respiration by mVOCs [36,37]. As a core component of mitochondrial Complex II, SDH links the TCA cycle to the electron transport chain. We propose that this increase reflects a compensatory metabolic adaptation to sustain basal bioenergetic flux under stress, rather than enhanced ATP production, and may serve to re-establish redox balance through modulation of the NAD^+^/NADH ratio. To investigate the molecular basis of the observed SDH activity increase, we examined the transcriptomic expression of genes encoding SDH subunits and related assembly factors (SDHA, SDHB, SDHF1, SDHF2, and SDHF3) in the M2M-treated group. The results are presented as a heatmap in Supplementary Figure S1. As shown, the expression of these genes exhibited some variation between the control and M2M-treated groups; however, none of these changes were statistically significant (|log_2_FC| < 1.5, padj ≥ 0.05) following M2M exposure. This suggests that the nearly twofold increase in SDH enzymatic activity is not attributable to transcriptional upregulation of SDH subunit genes. Instead, we propose that this increase reflects post-translational activation of the existing SDH complex, potentially through reversible redox modifications under oxidative stress, allosteric regulation by altered TCA cycle intermediates (e.g., citrate and isocitrate), or altered assembly of SDH within mitochondrial Complex II. Additionally, the significant increase in G6PDH activity after M2M treatment suggests the potential involvement of the pentose phosphate pathway in the response of *B. cinerea* to oxidative stress. G6PDH is the key rate-limiting enzyme in the oxidative phase of the pentose phosphate pathway. The NADPH it produces is not only involved in maintaining cellular reducing power but also provides electrons for the glutathione cycle and other antioxidant systems [38]. Previous studies have shown that changes in NADPH metabolism in *Aspergillus nidulans* affect the response to H_2_O_2_ stress and regulate peroxiredoxin-related processes [38]. Therefore, the M2M treatment induced changes in redox-related physiological regulation in *B. cinerea*, with antioxidant defense, respiratory metabolism, and reducing power supply likely jointly participating in the stress response. Direct ROS detection assays (e.g., DCFH-DA fluorescence for intracellular ROS, NBT staining for superoxide, or DAB staining for H_2_O_2_) were not performed in this study. Instead, we assessed oxidative stress through complementary indirect approaches: (i) the activities of key antioxidant enzymes (SOD, CAT, and POD), which are typically upregulated in response to increased intracellular ROS; (ii) the levels of MDA, a marker of lipid peroxidation; and (iii) the transcriptional profiling of ROS-scavenging genes. While direct ROS visualization would provide more immediate evidence of oxidative burst, the convergence of enzymatic, transcriptional, and physiological data collectively supports the interpretation that M2M treatment imposes an oxidative challenge on *B. cinerea*. Future studies incorporating DCFH-DA staining or quantitative H_2_O_2_ measurement would be valuable to further corroborate these findings.

Before further interpreting the molecular mechanisms, it should be acknowledged that the double-dish inverted culture method employed in this study is a semi-quantitative bioassay. The actual vapor concentration of M2M in the headspace was not directly measured, and whether true vapor-liquid equilibrium was established during the 5-day incubation period could not be determined. The EC_50_ value reported here (3.49 μL/plate, corresponding to approximately 53.7 μL/L headspace) is expressed based on the volume of M2M applied per plate, which is the standard practice for this assay system [10,11]. This approach allows for reproducible comparisons of antifungal activity under standardized conditions, but the results should be interpreted with the understanding that the actual biological exposure is determined by the headspace concentration rather than the applied volume. Nonetheless, the consistent experimental setup across all treatments ensures that the relative potency of M2M at different concentrations is reliably assessed.

Transcriptomic analysis revealed the molecular basis of the M2M-induced physiological responses in *B. cinerea*. After M2M treatment, DEGs were mainly enriched in pathways related to glyoxylate and dicarboxylate metabolism, pyruvate metabolism, carbon metabolism, and redox processes, while ribosome biogenesis, RNA processing, and protein synthesis-related processes were suppressed. This suggests that M2M stress is associated with alterations in central metabolism and redox regulation, and may also influence protein synthesis-related processes, thereby contributing to mycelial growth inhibition [28]. Consistent with the changes in physiological indicators, further analysis of ROS-related gene expression indicated that M2M significantly regulated the expression of antioxidant-related genes, including *CAT*, *POD*, *GPX*, *PRX*, and *GST*. The expression levels of some of these genes were significantly correlated with antioxidant enzyme activities and MDA content, suggesting that the M2M-induced redox response is not only manifested as changes in enzyme activities but is also accompanied by the regulation of ROS metabolism-related genes. Previous studies have shown that *B. cinerea* can maintain ROS homeostasis through transcriptional regulation. For example, the AP-1 transcription factor Bap1 regulates the expression of H_2_O_2_-induced ROS detoxification-related genes, participating in the oxidative stress response [39,40]. Deletion of *BcMsn2* led to increased ROS accumulation and affected the activities and related gene expression of antioxidant systems, including CAT and SOD [41]. Therefore, the changes in ROS-related gene expression and the enhanced antioxidant enzyme activities together indicate that M2M induced the activation of the redox defense response in *B. cinerea*.

Metabolomic analysis further revealed the impact of M2M on metabolic homeostasis in *B. cinerea*. M2M treatment significantly altered the levels of central metabolites, including citric acid, isocitrate, L-glutamic acid, L-glutamine, and L-aspartic acid, which are involved in the TCA cycle, amino acid metabolism, and carbon source conversion, suggesting that M2M may perturb the basal metabolic balance of *B. cinerea*. Furthermore, among the DAMs, lipids and lipid-like molecules emerged as the major class of altered metabolites after M2M treatment, with their proportion increasing from 54.68% to 72.96%. Lipids are not only essential components of the fungal cell membrane but are also involved in membrane stability regulation, signal transduction, and energy storage [42], and they influence fungal morphology, growth, stress adaptation, and pathogenicity [43]. Yue et al. demonstrated that microbial VOCs could inhibit mycelial growth by affecting membrane integrity and lipid-related metabolic processes in *B. cinerea* [44]. Therefore, the lipid-related metabolic changes in our study may be associated with the M2M-induced mycelial structural abnormalities.

Integrated transcriptomic and metabolomic analyses further indicated that several metabolic pathways in *B. cinerea* showed concurrent changes in gene expression and metabolite accumulation after M2M treatment. Co-enrichment analysis showed that pathways including carbon metabolism, glyoxylate and dicarboxylate metabolism, glycerophospholipid metabolism, and glutathione metabolism all contained both DEGs and DAMs. The correlations between lipid-related metabolite changes and related genes suggested that M2M treatment may affect lipid metabolic processes in *B. cinerea*. Additionally, the gene-metabolite correlations in carbon metabolism and redox-related pathways further indicated that the M2M-induced response in *B. cinerea* involves coordinated changes across multiple metabolic processes. However, it should be acknowledged that the correlation analysis between transcriptomic and metabolomic data was performed without multiple-testing correction. A total of 702 Spearman correlation pairs were tested, and significance was determined using unadjusted *p*-values (|*r*| > 0.75, *p* < 0.05). Although this exploratory approach may be susceptible to false-positive associations, the combination of a stringent correlation coefficient threshold (|*r*| > 0.75) and the fact that the identified correlations were largely consistent with the known biological functions of the involved genes and metabolites supports the biological relevance of these associations. Future studies employing functional validation (e.g., gene knockout or pharmacological inhibition) would be valuable to confirm the causal relationships suggested by these correlations.

It should also be noted that the antifungal activity of M2M against *B. cinerea* was evaluated exclusively in vitro using the double-dish inverted culture system. While this approach provides a standardized and reproducible platform for assessing the intrinsic antifungal activity of volatile compounds and for elucidating the underlying physiological and molecular responses, it does not fully replicate the complex environmental conditions encountered during postharvest storage of fresh produce. Several aspects remain to be evaluated before M2M can be considered for practical application: (i) its efficacy against *B. cinerea* infection on intact or wounded fruit surfaces; (ii) its potential phytotoxicity to host fruit tissues; (iii) its residue behavior and dissipation dynamics under storage conditions; and (iv) its performance under varying temperature, humidity, and packaging conditions typical of commercial storage. Future studies incorporating fruit-based infection models and storage trials will be necessary to determine the practical feasibility of M2M as a postharvest fumigant.

## 4. Materials and Methods

### 4.1. Fungal Strain and Culture Conditions

The *B. cinerea* strain used in this study was originally isolated from diseased blueberry fruits and kindly provided by a collaborating laboratory. The strain was authenticated by morphological observation and molecular identification based on internal transcribed spacer (ITS) region sequencing using the primers ITS1 and ITS4 according to White et al. [45]. The ITS sequence has been deposited in GenBank under accession number PZ944795. The strain was maintained on potato dextrose agar (PDA) at 4 °C.

Methyl 2-methylbutyrate (M2M, ≥99.5% purity) was procured from Macklin Biochemical Co., Ltd. (Shanghai, China). Commercial assay kits for quantifying the activities of glucose-6-phosphate dehydrogenase (G6PDH), superoxide dismutase (SOD), catalase (CAT), and peroxidase (POD) were purchased from Ensi Biological Technology Co., Ltd. (Shanghai, China).

PDA medium was prepared with the following composition: 200 g of potato extract, 20 g of glucose, 15–20 g of agar, and deionized water to a final volume of 1 L. The pH was adjusted to 5.5–6.0 prior to autoclaving at 121 °C for 20 min.

In all experiments, three independent biological replicates were performed for antifungal activity assays, physiological and biochemical analyses, and transcriptome sequencing, while six independent biological replicates were used for untargeted metabolomics. For each biological replicate, the culture was independently established from a separate mycelial plug taken from the same mother plate, and the entire experimental procedure was carried out independently for each replicate. Technical replicates (three technical repeats per biological replicate) were included in physiological and biochemical assays to ensure measurement precision.

### 4.2. Assessment of Antifungal Activity

The antifungal activity of M2M against *B. cinerea* was evaluated using a modified double-dish inverted culture system [10]. Briefly, 20 mL of molten PDA was poured into the bottom of a sterile 90 mm Petri dish. Once solidified, a 5 mm mycelial plug, taken from the margin of an actively growing colony, was centrally inoculated onto the agar surface. Subsequently, 10 mL of PDA was dispensed into the lid of the dish, and 0 (control), 3, 5, 7, 10, or 15 μL of M2M was pipetted onto the agar surface in the lid. The dishes were immediately inverted to prevent direct liquid contact between the volatile source and the fungal colony, and the edges were sealed with parafilm to minimize volatile loss. All treatments were incubated in the dark at 25 °C for 5 days, with three independent biological replicates per concentration (each replicate consisted of a separately prepared culture dish inoculated with a mycelial plug from the same mother plate).

Colony diameters were measured via the cross-bisector method. The mycelial growth inhibition rate (%) was calculated using the following formula:

$$mycelial\;growth\;inhibition\;\left(\%\right)=\frac{C-T}{C}\times 100$$

where represents the mean colony diameter (mm) in the control group and represents the mean diameter in the M2M-treated group. The half-maximal effective concentration (EC_50_) was calculated using linear regression analysis of the dose–response data. Briefly, the percentage inhibition values were converted to probit units, and the M2M concentrations were log_10_-transformed prior to analysis. The regression equation was derived by plotting the probit-transformed inhibition rates against the log_10_-transformed concentrations. The EC_50_ was then calculated by solving the regression equation for the concentration corresponding to 50% inhibition (probit = 5). All analyses were performed using SPSS software (version 26.0; IBM Corp., Armonk, NY, USA). The EC_50_ value was expressed based on the volume of M2M applied per plate (μL/plate), as the actual vapor concentration in the sealed Petri dish cannot be directly measured in the double-dish inverted culture system. This approach is consistent with previous studies using the same experimental system [10,11,28]. The internal volume of the 90 mm Petri dish used in this study was approximately 95 mL, with a headspace volume of approximately 65 mL after adding 30 mL of PDA medium (bottom: 20 mL; lid: 10 mL). Based on this headspace volume, the EC_50_ of 3.49 μL/plate corresponds to approximately 53.69 μL/L of headspace. All treatments were incubated at a constant temperature of 25 °C to ensure consistent volatilization conditions across all concentrations. The dishes were immediately inverted and sealed with Parafilm to minimize volatile loss and to ensure that the M2M vapor was retained within the closed headspace. Although the actual volatilization losses were not quantified, the standardized experimental conditions (identical dish size, medium volume, sealing method, and incubation temperature) ensured consistent exposure across all treatments, allowing for reliable comparative analysis.

### 4.3. Scanning Electron Microscopy (SEM) Observation

To observe M2M-induced ultrastructural alterations, *B. cinerea* mycelia were treated with 3 μL/plate M2M (a concentration approximately corresponding to the calculated EC_50_ of 3.49 μL/plate), while sterile water served as the control. After 5 days of incubation, mycelial fragments were carefully excised and affixed to aluminum stubs. Samples were rapidly cryo-fixed by immersion in liquid nitrogen for 2 min and subsequently transferred to the preparation chamber of a cryo-SEM (Sigma 300, ZEISS, Jena, Germany). Sublimation drying was conducted at −100 °C for 15 min. The cold stage was maintained at −140 °C throughout the process. Dried specimens were sputter-coated with gold for 120 s (60 s × 2). High-resolution micrographs were acquired using the integrated imaging system [27].

### 4.4. Physiological and Biochemical Analyses

M2M treatment was performed according to the culture system described in Section 4.2. For physiological analyses, the M2M concentration was set at 3 μL/plate (approximately the EC_50_). After 5 days of incubation, mycelial samples from the M2M-treated and control groups were collected, frozen immediately in liquid nitrogen, and stored at −80 °C for later use. The activities of G6PDH, SOD, CAT, and POD in the mycelial samples were determined using commercial kits according to the manufacturers’ instructions. Additionally, the malondialdehyde (MDA) content and the activities of succinate dehydrogenase (SDH) and lactate dehydrogenase (LDH) were measured using established methods. All assays were performed with three biological replicates. All assays were performed with three biological replicates. For each biological replicate, technical triplicates were included in the spectrophotometric measurements to ensure accuracy; the mean value of the technical triplicates was used as the representative value for that biological replicate.

### 4.5. Transcriptome Sequencing and Data Analysis

Samples for transcriptome analysis were prepared using the double-dish inverted culture system with 3 μL/plate M2M. After 5 days of incubation, *B. cinerea* mycelia were collected, frozen in liquid nitrogen, and stored at −80 °C. Three biological replicates were included for each group: the M2M-treated group (Y1–Y3) and the control group (CK1–CK3). Each biological replicate was derived from an independently treated culture dish, not from aliquots of the same culture. RNA extraction, library construction, and sequencing were performed individually for each biological replicate. RNA extraction, library construction, and sequencing were performed by Novogene Co., Ltd. (Beijing, China).

Total RNA was extracted using the TRIzol method. RNA concentration, purity, and integrity were assessed using a NanoDrop 2000 spectrophotometer (Thermo Fisher Scientific, Wilmington, DE, USA), agarose gel electrophoresis, and an Agilent 2100 Bioanalyzer (Agilent Technologies, Santa Clara, CA, USA). mRNA was enriched using Oligo (dT) magnetic beads, followed by RNA fragmentation, first-strand and second-strand cDNA synthesis, end repair, A-tailing, adapter ligation, and PCR amplification to construct the sequencing library. After quality control, the libraries were sequenced on an Illumina NovaSeq 6000 platform (Illumina, Inc., San Diego, CA, USA) to generate paired-end reads. Raw sequencing data were quality-controlled using fastp software (v0.23.1) [46] to remove adapter sequences, reads with a high proportion of ambiguous bases (N), and low-quality reads, resulting in clean reads. The clean reads were aligned to the *B. cinerea* reference genome (*B. cinerea* ASM83294v1.42, GCA_000143535.4) using HISAT2 (v2.2.1) software [47]. Gene identifiers (e.g., Bcin05g04580, Bcin06g04520, Bcin11g00390, Bcin11g03930, Bcin10g02270, Bcin08g00300) and their corresponding functional annotations were retrieved from the *B. cinerea* reference genome annotation (ASM83294v1.42, GCA_000143535.4) via the NCBI Genome database. All gene names and abbreviations used in this study follow the standard nomenclature from this reference annotation. Gene expression levels were quantified using featureCounts (v2.0.6) [48], generating a raw integer read count matrix for each gene across all samples. This raw count matrix was used as the input for differential expression analysis. Differentially expressed genes (DEGs) between the two groups were identified using DESeq2 (v1.42.0) software [49] with the criteria |log_2_Fold Change| > 1.5 and unadjusted *p* < 0.05. FPKM (fragments per kilobase of transcript per million mapped reads) values were calculated separately and used only for descriptive purposes (e.g., visualization of gene expression levels), and were not used as input for DESeq2. The unadjusted *p*-values were used for initial screening to identify candidate pathways for further integrated analysis with metabolomics data. For transparency, the adjusted *p*-values (padj) for all DEGs are provided in Supplementary Table S3. Gene Ontology (GO) functional enrichment and Kyoto Encyclopedia of Genes and Genomes (KEGG) pathway enrichment analyses were performed using the clusterProfiler (v4.8.1) package [50] in R.

### 4.6. Untargeted Metabolomics Analysis

Samples for metabolomics analysis were prepared using the same culture system as for transcriptomics, with 3 μL/plate M2M. Six biological replicates were included for each group: the M2M-treated group (T1–T6) and the control group (CK1–CK6). Each biological replicate was independently cultured and processed. Technical replicates were not performed for LC-MS analysis; instead, quality control (QC) samples were used to monitor instrument stability and reproducibility, as described above. After 5 days of incubation, mycelial samples were collected, frozen in liquid nitrogen, and stored at −80 °C. These samples were obtained from the same batch of independently treated cultures used for transcriptome sequencing (Section 4.5). For metabolite extraction, 100 mg of frozen, ground mycelial tissue was accurately weighed and extracted with 500 μL of pre-cooled 80% methanol in water. After thorough mixing, samples were incubated on ice for 5 min and then centrifuged at 15,000 g and 4 °C for 20 min. An appropriate amount of supernatant was taken and diluted with mass spectrometry-grade water to a final methanol concentration of 53%. The solution was centrifuged again at 15,000 g and 4 °C for 20 min, and the supernatant was collected for LC-MS analysis. Blank samples were used to remove background ion interference, and quality control (QC) samples, prepared by mixing equal volumes of all samples, were used to monitor instrument stability.

Metabolite detection was performed using an ultra-high-performance liquid chromatography-high-resolution mass spectrometry (UHPLC-HRMS) system. Chromatographic separation was achieved on a Hypersil Gold C18 column (2.1 mm × 100 mm, 1.8 μm) at a column temperature of 40 °C and a flow rate of 0.2 mL/min. Mobile phase A was water containing 0.1% formic acid, and mobile phase B was methanol, using a gradient elution program. Mass spectrometry was performed using an electrospray ionization (ESI) source in both positive and negative ion modes, with a mass scan range of *m*/*z* 100–1500. Raw MS data were converted to mzXML format using ProteoWizard and then processed using XCMS software (v4.0) for peak extraction, alignment, and area calculation. Metabolite identification was performed by matching retention times, accurate masses, and tandem mass spectrometry (MS/MS) data against the Novogene in-house database (NovoMetDB). Blank samples were used to remove background ions, and data were normalized. Metabolites with a coefficient of variation (CV) > 30% in QC samples were removed to obtain the final metabolite quantitative data.

Identified metabolites were annotated using the KEGG, HMDB, and LIPID MAPS databases. Multivariate statistical analyses, including principal component analysis (PCA) and orthogonal partial least squares discriminant analysis (OPLS-DA), were performed using the metaX software (v1.4.16) package. Differentially accumulated metabolites (DAMs) were selected based on VIP > 1, *p* < 0.05, and |log_2_FC| > 1.

### 4.7. Correlation Analysis

Spearman’s rank correlation analysis was used to explore potential associations between gene expression levels and physiological indicators, as well as between gene expression and metabolite accumulation. Correlations between the expression of ROS-related genes and the activities of antioxidant enzymes (SOD, CAT, POD) and MDA content in *B. cinerea* were analyzed.

For the integrated transcriptomic and metabolomic analysis, Spearman correlations between DEGs and DAMs in key metabolic pathways were calculated using the averaged log_2_FC values for each group (mean of 3 transcriptomic replicates and mean of 6 metabolomic replicates per group). A total of 702 correlation pairs were tested between the identified DEGs and DAMs within the co-enriched KEGG pathways. This approach was adopted because the two datasets were obtained from the same batch of independently treated cultures but with different numbers of biological replicates (*n* = 3 for transcriptomics, *n* = 6 for metabolomics), as detailed in Section 4.5 and Section 4.6. Significance was determined using unadjusted *p*-values with the criteria |*r*| > 0.75 and *p* < 0.05. No multiple-testing correction (e.g., FDR) was applied, as this analysis was intended as an exploratory tool to generate hypotheses and identify potential gene–metabolite associations at the network level, rather than as a confirmatory test for specific pairwise interactions. The significant gene-metabolite correlations were visualized as a network using Cytoscape software (version 3.9.1).

### 4.8. Quantitative Real-Time PCR (qRT-PCR) Validation

To validate the reliability of the RNA-seq data, a subset of DEGs was randomly selected for qRT-PCR validation using the same RNA samples (collected after 5 days of incubation) as those used for transcriptome sequencing. First-strand cDNA was synthesized from RNA using a reverse transcription kit in a 20 μL reaction volume containing 500 ng of RNA template. The reaction conditions were 42 °C for 30 min and 85 °C for 5 s. The resulting cDNA was stored at −80 °C.

qRT-PCR was performed using SYBR Green fluorescent dye. Each reaction was performed with three technical replicates per biological sample, and the mean Ct value was used for analysis. Standard curves for all primer pairs showed R^2^ > 0.98, indicating high amplification efficiency and linearity. Amplification curves were normal for all reactions, and positive and negative controls were included in each run to ensure reaction validity. Formal primer efficiency calculations and melt-curve analysis were not performed in this study; nevertheless, the standard curves (R^2^ > 0.98), normal amplification curves, and inclusion of positive and negative controls confirmed the specificity and reliability of the amplification. The Actin gene was used as the internal reference. The stability of Actin expression was verified by comparing raw Ct values between the control and M2M-treated groups (CK: 25.66 ± 0.82; Y: 25.97 ± 1.32; *p* = 0.37), confirming no significant difference in Actin expression across treatments. The reaction mixture (16 μL total volume) contained 8 μL of 2 × SYBR Premix, 2 μL of cDNA template, 0.32 μL each of forward and reverse primers (10 μmol/L), and 5.36 μL of ddH_2_O. The amplification program consisted of an initial denaturation at 95 °C for 10 min, followed by 40 cycles of 95 °C for 15 s and 60 °C for 30 s. The *Actin* gene was used as the internal reference, and relative gene expression levels were calculated using the 2^−^ΔΔCt method. Primers specific to the genes used for qRT-PCR validation are listed in Supplementary Table S3.

### 4.9. Data Statistics and Analysis

All experimental data are presented as the mean ± standard error (SE). The effect of different M2M concentrations on the mycelial growth inhibition rate was analyzed using one-way analysis of variance (ANOVA) followed by Duncan’s multiple range test for significance. For comparisons between the CK and M2M-treated groups, Welch’s two-sample *t*-test was applied, as the assumption of equal variances was not satisfied for some parameters (Levene’s test, *p* < 0.05). The assumptions of normality were verified using the Shapiro–Wilk test. A *p*-value < 0.05 was considered statistically significant.

Transcriptomic and metabolomic data analysis and visualization were performed using R software (version 4.4.2).

## 5. Conclusions

This study systematically evaluated the antifungal activity of the microbial volatile organic compound (mVOC) methyl 2-methylbutyrate (M2M) against *Botrytis cinerea* and characterized the induced physiological and molecular responses. The results demonstrated that M2M possesses strong antifungal activity, significantly affecting the normal growth and morphological structure of *B. cinerea* mycelia, and inducing changes in redox status and metabolic levels. Multi-omics analyses further revealed that M2M treatment was associated with extensive adjustments in gene expression and metabolite accumulation patterns in *B. cinerea*, involving central carbon metabolism, lipid metabolism, redox regulation, and related transcriptional regulatory processes. Integrated transcriptomic and metabolomic analyses showed that several metabolic processes exhibited responses at both the gene expression and metabolite accumulation levels, further revealing the multi-layered regulatory changes in *B. cinerea* in response to M2M stress. In conclusion, M2M treatment was associated with comprehensive morphological, physiological, and molecular responses in *B. cinerea* and exhibited a marked antifungal effect. This study provides mechanistic insights and foundational data supporting the potential of M2M as a candidate for the development of volatile-based control strategies against gray mold disease. However, further studies that involve fruit infection models and storage trials are required to evaluate its practical applicability and safety as a postharvest fumigant.

## Figures and Tables

**Figure 1:**
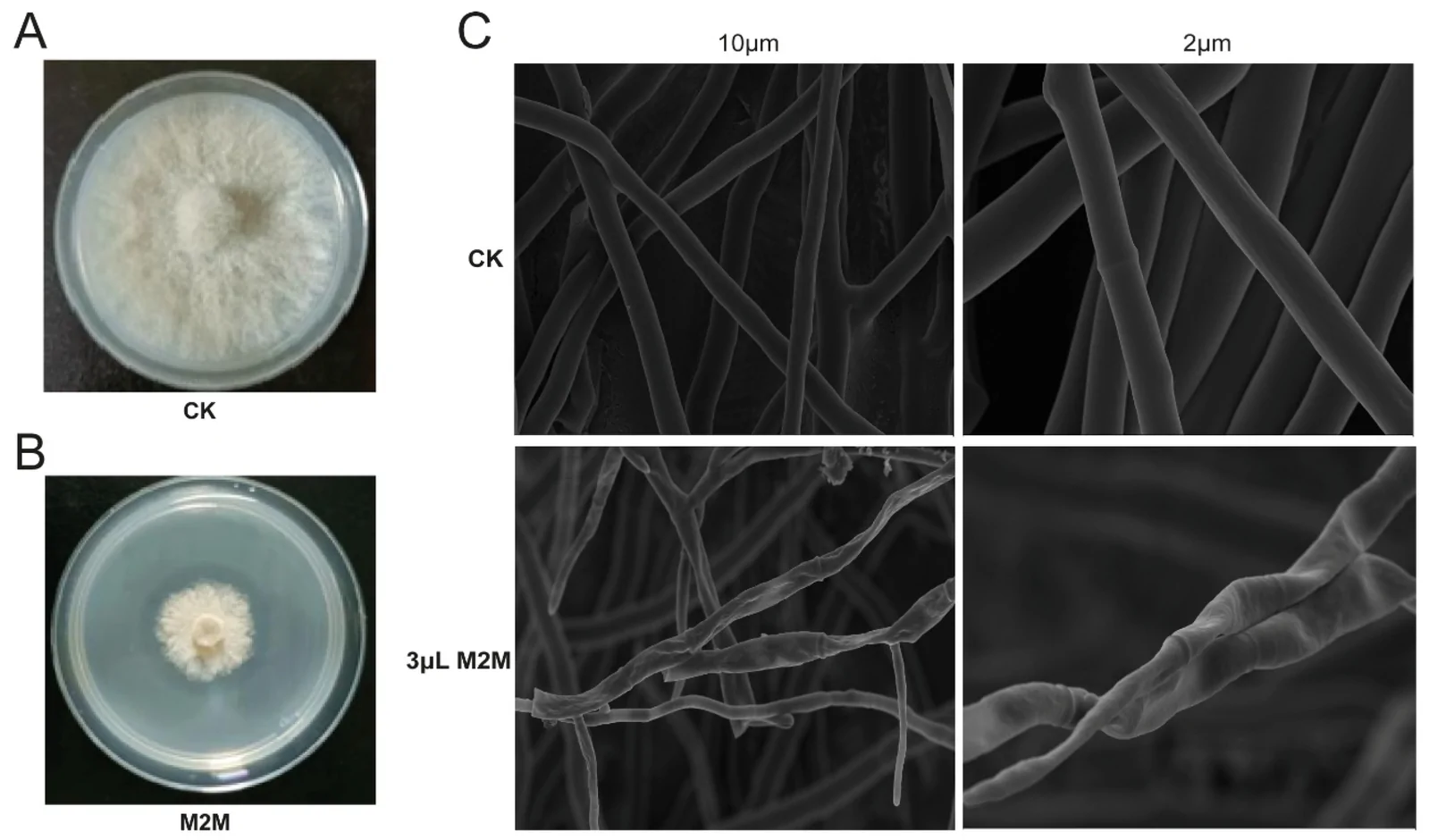
Methyl 2-methylbutyrate (M2M) inhibits mycelial growth and induces ultrastructural abnormalities in *Botrytis cinerea*. (**A**,**B**) Colony morphology of *B. cinerea* after M2M treatment. (**A**) control (0 μL/plate); (**B**) 5 μL/plate M2M treatment group. (**C**) Scanning electron microscopy images showing ultrastructural changes in *B. cinerea* mycelia after M2M treatment (3 μL/plate). Scale bars: 10 μm (**left column**) and 2 μm (**right column**) in (**C**). (**A**,**B**) are colony photographs on 90-mm Petri dishes and are shown without scale bars.

**Figure 2:**
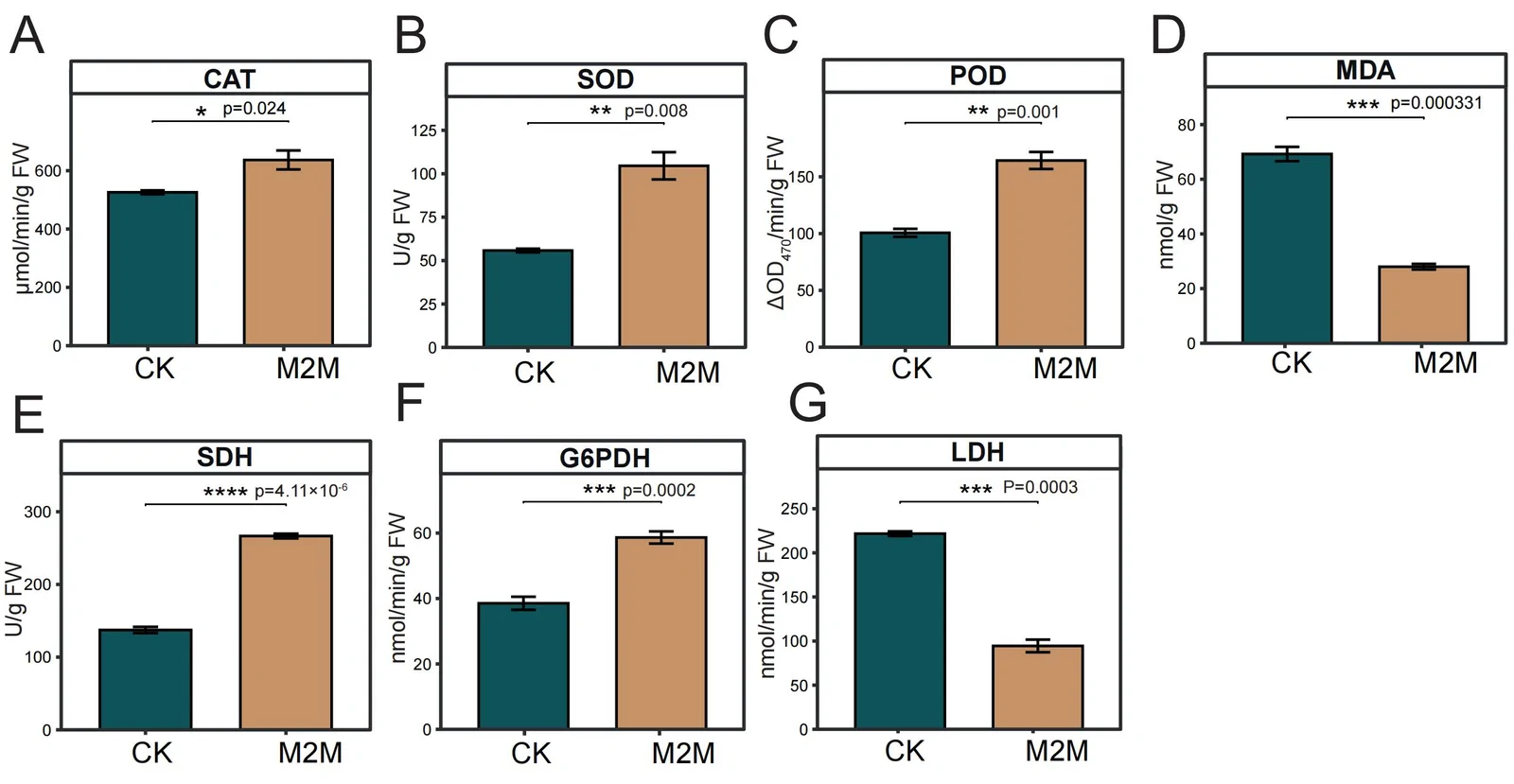
Effect of methyl 2-methylbutyrate (M2M) treatment on physiological and biochemical indicators of *B. cinerea*. (**A**) Catalase (CAT) activity. (**B**) Superoxide dismutase (SOD) activity. (**C**) Peroxidase (POD) activity. (**D**) Malondialdehyde (MDA) content. (**E**) Succinate dehydrogenase (SDH) activity. (**F**) Glucose-6-phosphate dehydrogenase (G6PDH) activity. (**G**) Lactate dehydrogenase (LDH) activity. Data are shown as mean ± SE (*n* = 3). Significance between the two groups was analyzed by Welch’s *t*-test. Exact *p*-values are shown above each comparison: * *p* < 0.05, ** *p* < 0.01, *** *p* < 0.001, **** *p* < 0.0001. Statistical summaries, including effect sizes (Cohen’s d) and 95% confidence intervals, are provided in Table 2.

**Figure 3:**
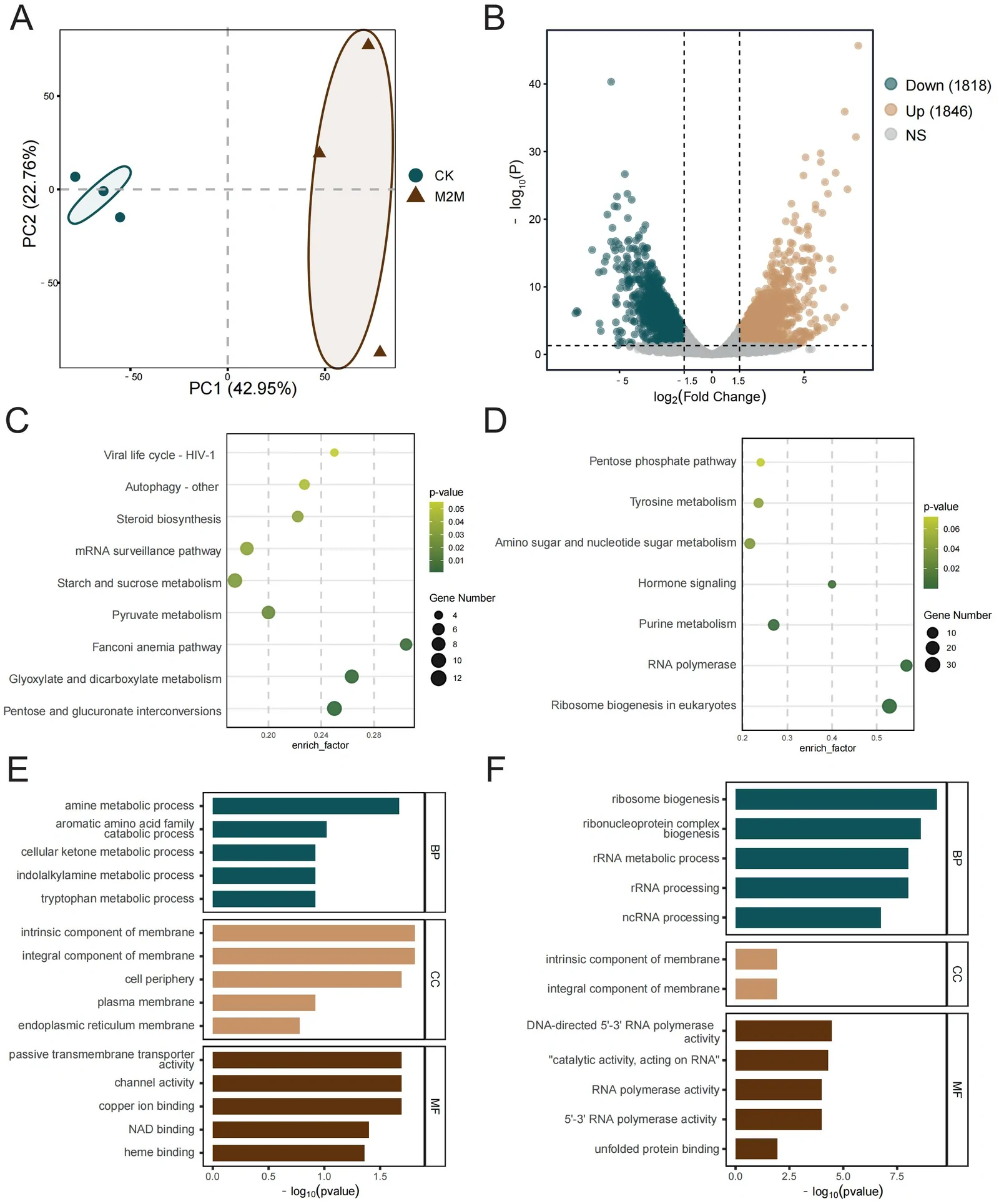
M2M treatment induces transcriptomic changes and functional enrichment in *B. cinerea*. (**A**) Principal component analysis (PCA) of samples from the control group (CK) and the M2M-treated group (Y). (**B**) Volcano plot of differentially expressed genes (DEGs) (|log_2_Fold Change| > 1.5 and *p* < 0.05). (**C**) KEGG pathway enrichment analysis of upregulated DEGs. (**D**) KEGG pathway enrichment analysis of downregulated DEGs. (**E**) GO functional enrichment analysis of upregulated DEGs. (**F**) GO functional enrichment analysis of downregulated DEGs.

**Figure 4:**
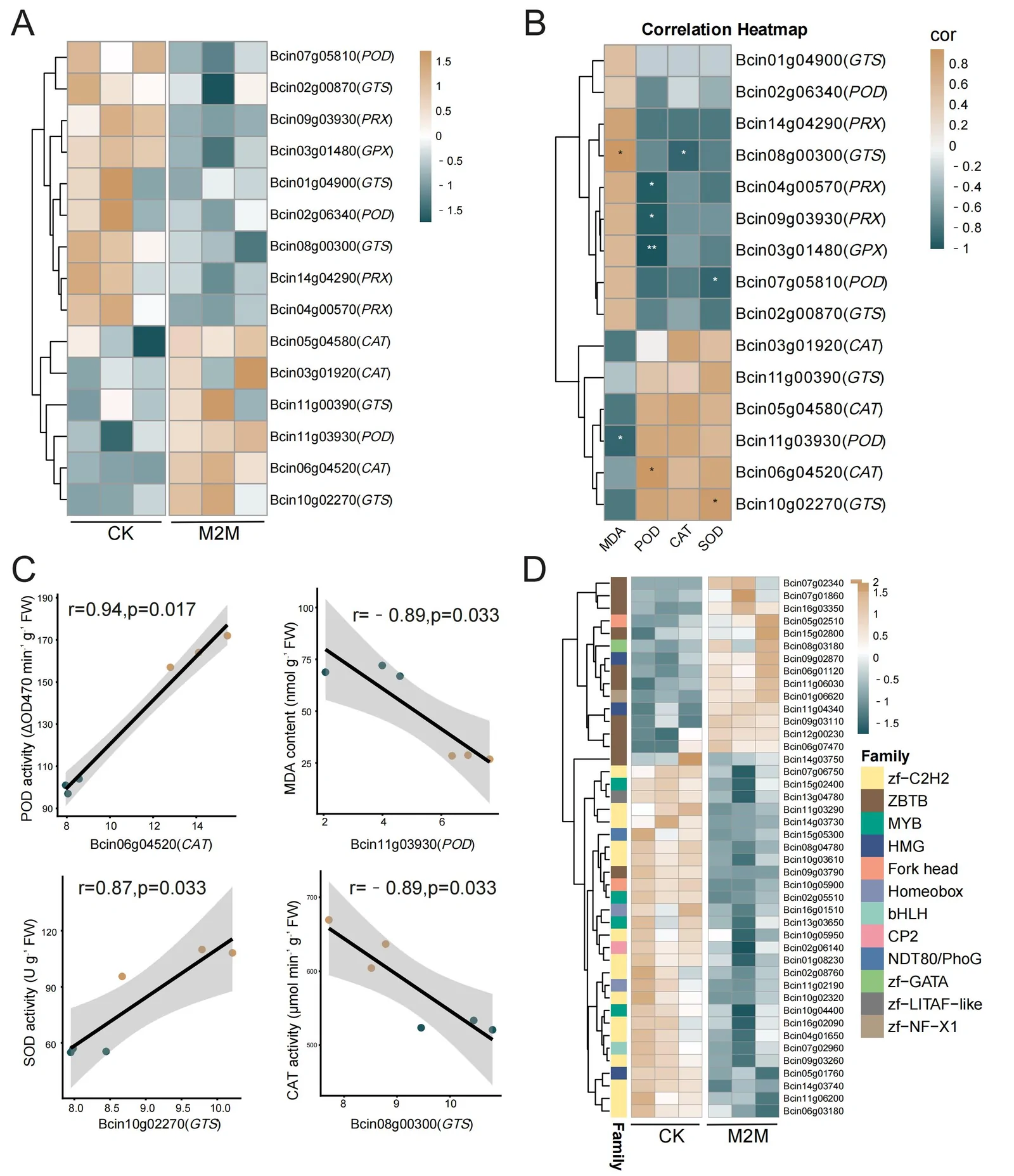
M2M treatment induces changes in ROS-related gene expression and transcription factor response in *B. cinerea*. (**A**) Heatmap of ROS metabolism-related DEGs in the M2M-treated group and the control group. (**B**) Spearman correlation analysis between the expression levels of ROS-related genes and antioxidant enzyme activities and MDA content. (**C**) Spearman correlation scatter plots for representative ROS-related genes and oxidative stress indicators. (**D**) Heatmap of differentially expressed transcription factors involved in the M2M response, with family classification. * *p* < 0.05, ** *p* < 0.01.

**Figure 5:**
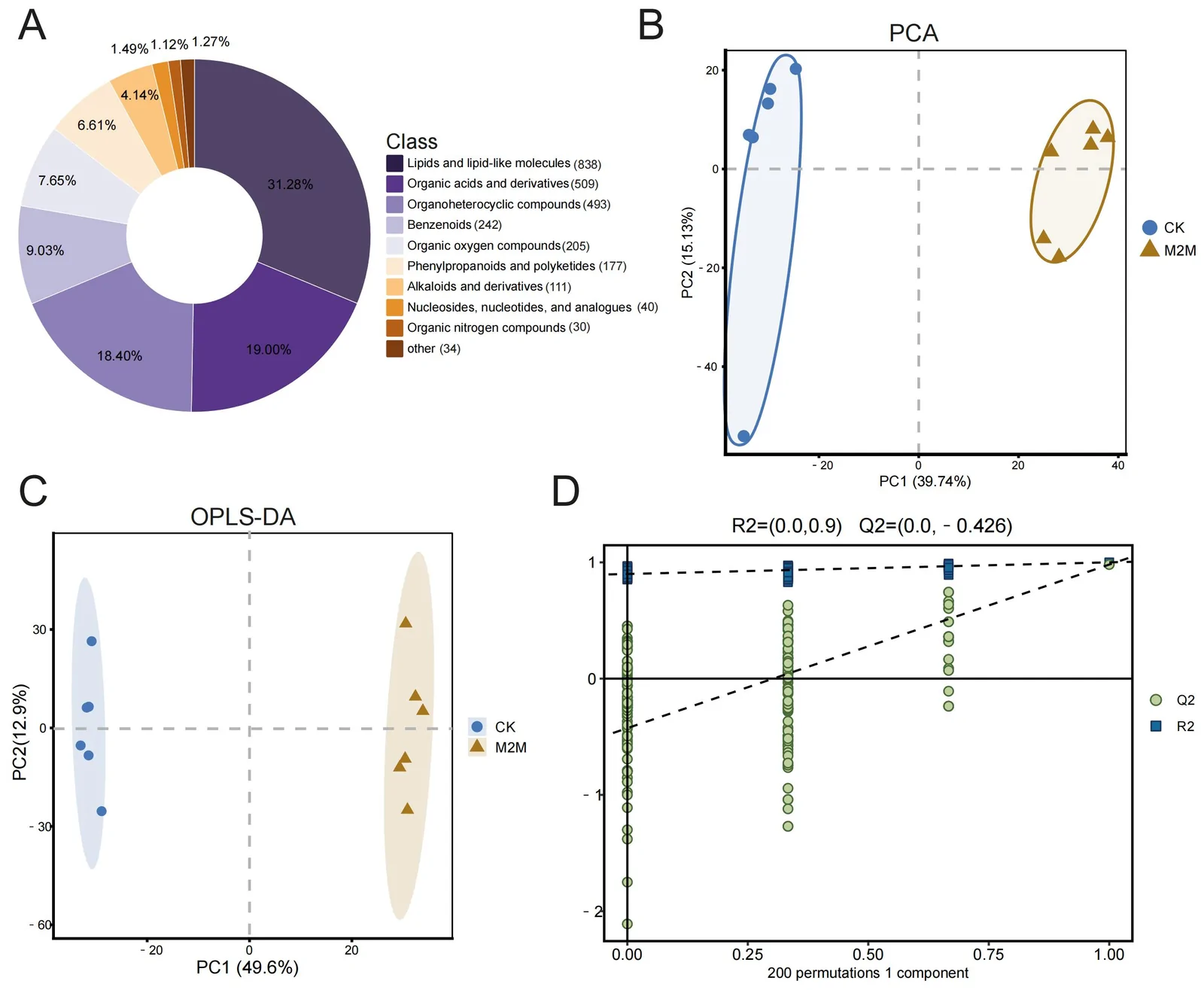
Global metabolic profile analysis of *B. cinerea* after M2M treatment. (**A**) Classification statistics of metabolites identified by untargeted metabolomics. (**B**) Principal component analysis (PCA) of the metabolic profiles of the M2M-treated and control groups. (**C**) OPLS-DA based on untargeted metabolomics data. (**D**) 200-fold permutation test of the OPLS-DA model.

**Figure 6:**
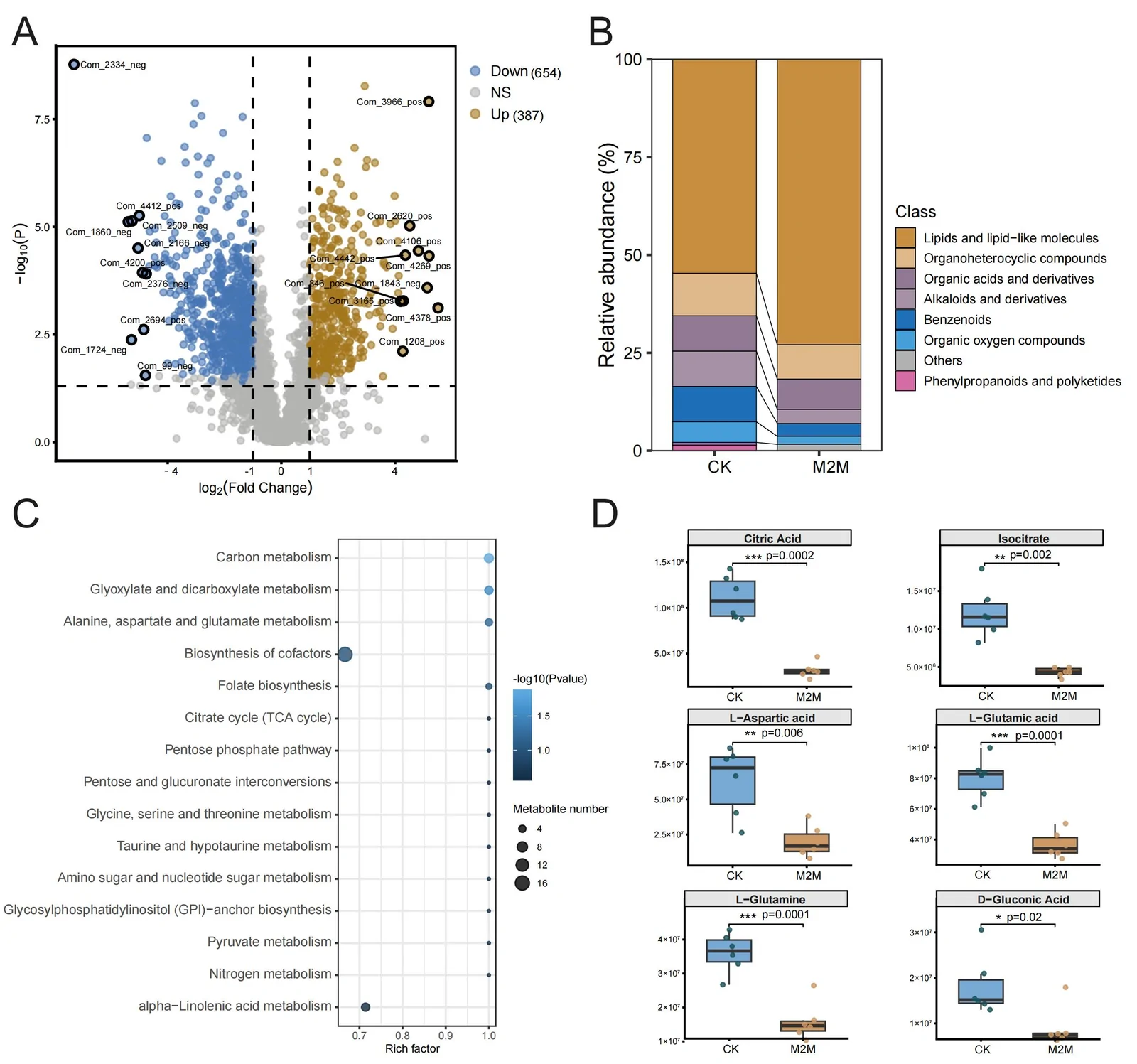
M2M treatment induces changes in DAM accumulation and metabolic pathways in *B. cinerea*. (**A**) Volcano plot of DAMs between the M2M-treated group (M2M) and the control group (CK), with DAMs selected based on VIP > 1, *p* < 0.05, and |log_2_FC| > 1. (**B**) Comparison of the chemical class composition of DAMs between the CK and M2M-treated groups. (**C**) KEGG pathway enrichment analysis of DAMs. (**D**) Changes in the levels of key differential metabolites. Data are shown as mean ± SE (*n* = 6). Significance between the two groups was analyzed by Welch’s *t*-test. Exact *p*-values are shown above each comparison: * *p* < 0.05, ** *p* < 0.01, *** *p* < 0.001.

**Figure 7:**
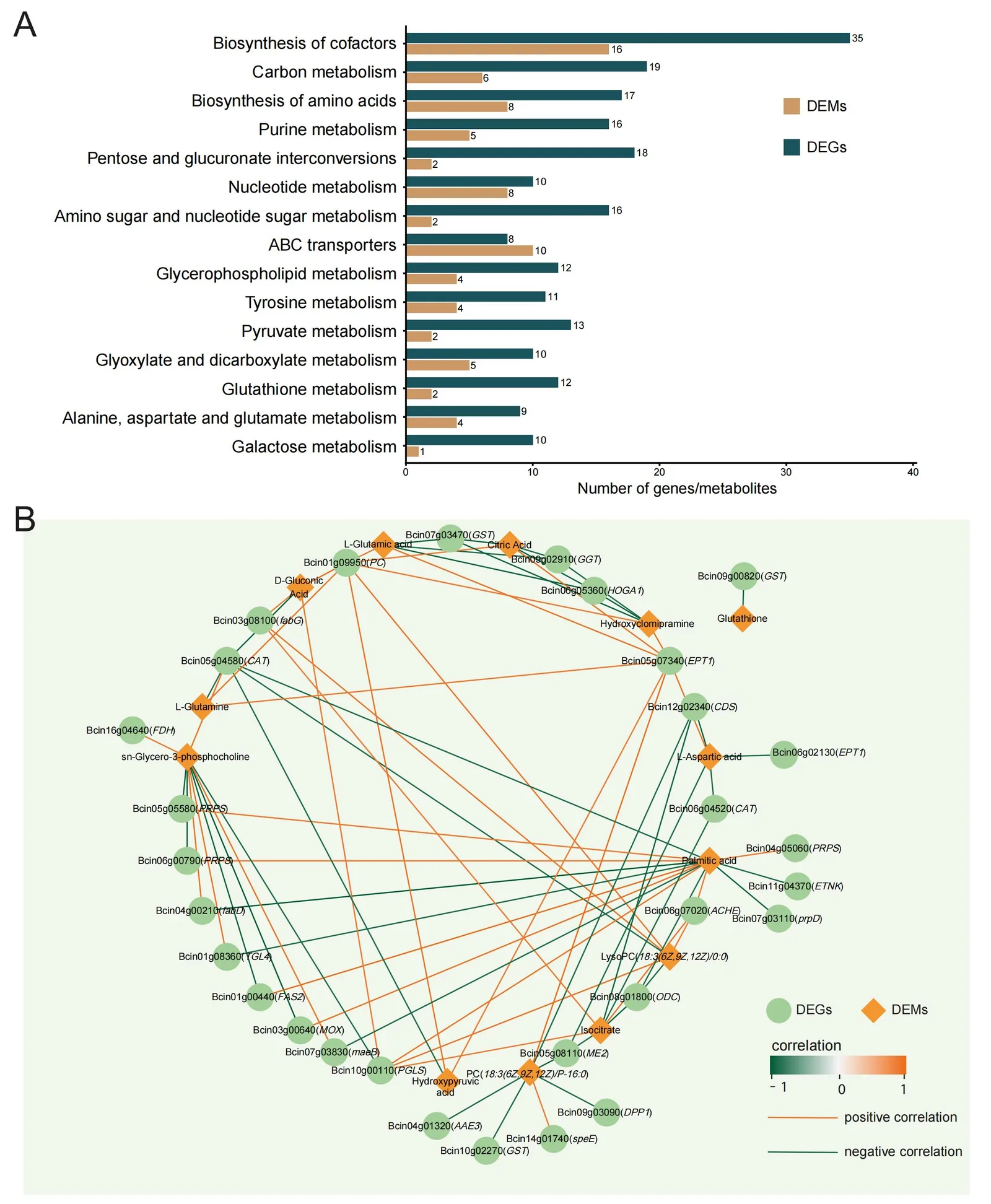
Integrated transcriptomic and metabolomic analysis reveals M2M-responsive metabolic pathways. (**A**) KEGG pathway co-enrichment analysis of DEGs and DAMs, showing the top 15 co-enriched metabolic pathways. (**B**) Spearman correlation network of DEGs and DAMs in key metabolic pathways. Gene-metabolite associations were selected based on a correlation coefficient (|*r*| > 0.75) and significance (*p* < 0.05). Nodes represent metabolites or genes, and edges represent significant correlations.

**Figure 8:**
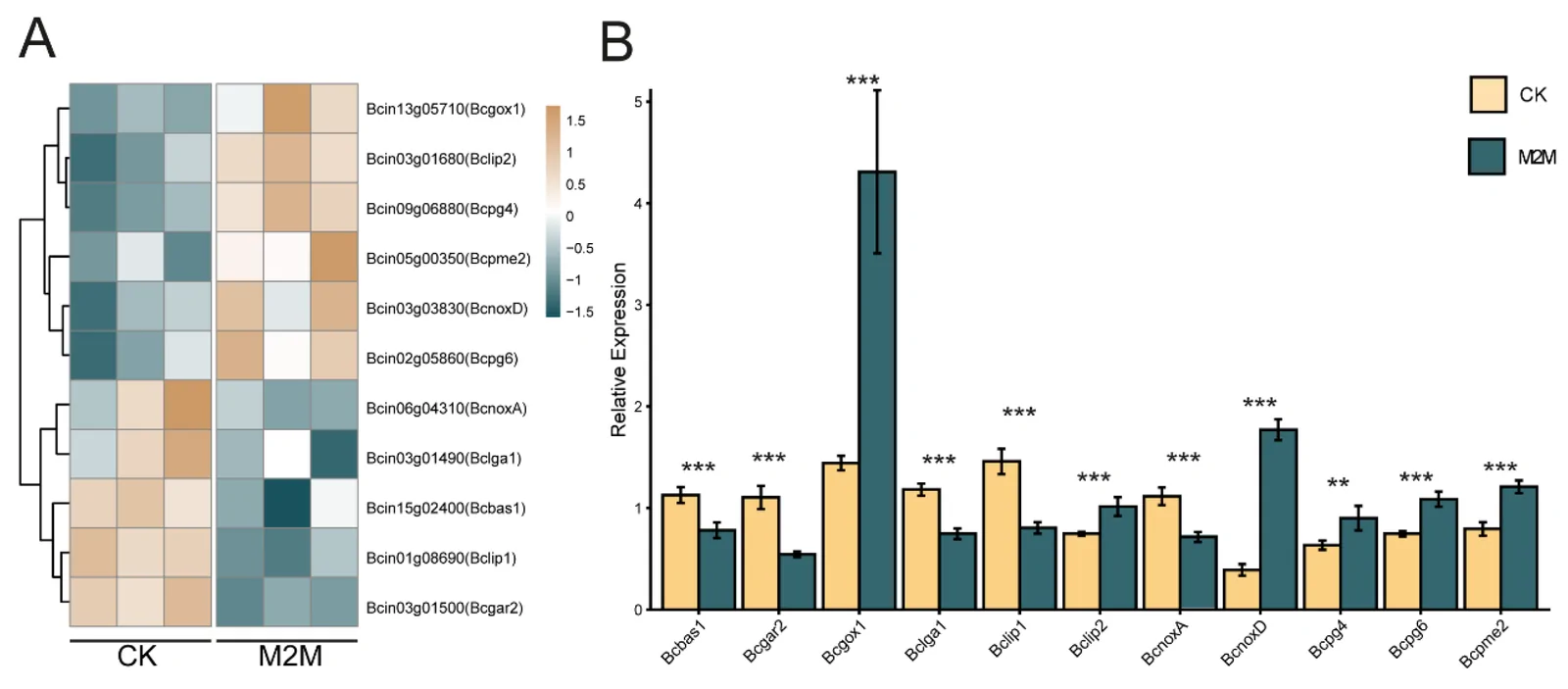
qRT-PCR validation of transcriptome sequencing results. (**A**) Heatmap of the expression patterns of selected DEGs. (**B**) qRT-PCR validation results for 10 candidate genes. Data are shown as mean ± SE (*n* = 3). Significance between the two groups was analyzed by Student’s *t*-test. ** *p* < 0.01, *** *p* < 0.001.

**Table 1:** Antifungal activities of methyl 2-methylbutyrate against mycelial growth of *Botrytis cinerea*.

Compound	Regression Equation	R^2^	EC_50_ (μL/Plate)	95% Confidence Limits (μL/Plate)
M2M	y = 3.08x − 1.67	0.77	3.49	3.018~3.906

Note: The regression equation was derived from probit-transformed inhibition rates (y) and log_10_-transformed M2M concentrations (x). EC_50_ was calculated as the concentration corresponding to probit = 5 (50% inhibition). The EC_50_ value is expressed based on the volume of M2M applied per plate (μL/plate), not the actual vapor concentration. Based on a headspace volume of approximately 65 mL, EC_50_ of 3.49 μL/plate corresponds to approximately 53.69 μL/L of headspace.

**Table 2:** Physiological parameters of *B. cinerea* in control (CK) and M2M-treated (Y) groups.

Parameter	CK (Mean ± SE)	Y (Mean ± SE)	Change	Cohen’s d	95% CI of Difference
CAT (μmoL/min/g)	525.86 ± 3.13	636.78 ± 15.34	+21.1%	5.79	(67.48, 154.36)
SOD (U/g)	55.79 ± 0.49	104.56 ± 3.79	+87.4%	10.42	(38.16, 59.38)
POD (△OD470/min/g)	100.67 ± 1.67	164.33 ± 3.54	+63.3%	13.29	(52.81, 74.51)
MDA (nmol/g)	69.23 ± 1.24	27.99 ± 0.50	−59.6%	25.15	(37.52, 44.96)
SDH (U/g)	137.36 ± 2.02	266.64 ± 1.33	+94.1%	43.68	(122.56, 136.00)
G6PDH nmol/min/g	38.54 ± 0.94	58.64 ± 0.88	+52.2%	12.80	(16.55, 23.65)
LDH nmol/min/g	221.73 ± 1.22	94.48 ± 3.29	−57.4%	29.59	(117.51, 136.99)

Note: Data are presented as mean ± SE (*n* = 3). *p*-values were calculated using Welch‘s two-sample *t*-test after verifying assumptions of normality (Shapiro–Wilk test). Effect sizes (Cohen’s d) were calculated using pooled standard deviation; all values > 0.8 indicate large treatment effects. Individual replicate values for all parameters are provided in Supplementary Table S1.

## Data Availability

No new data were created or analyzed in this study. Data sharing is not applicable to this article.

## Supplementary Materials

The following supporting information can be downloaded at: https://www.mdpi.com/article/10.3390/ijms27198589/s1.
