# Supplementary material for: Antifungal Activity of Methyl 2-Methylbutyrate Against Botrytis cinerea and the Induced Physiological, Transcriptional, and Metabolic Responses
Source: Int J Mol Sci. 2026 Sep 25;27(19):8589. doi: 10.3390/ijms27198589 (PMC13665467; doi:10.3390/ijms27198589)
Supplement: Supplementary file 1 [file ijms-27-08589-s001.zip › Figure S1 SDH gene heatmap.pdf]

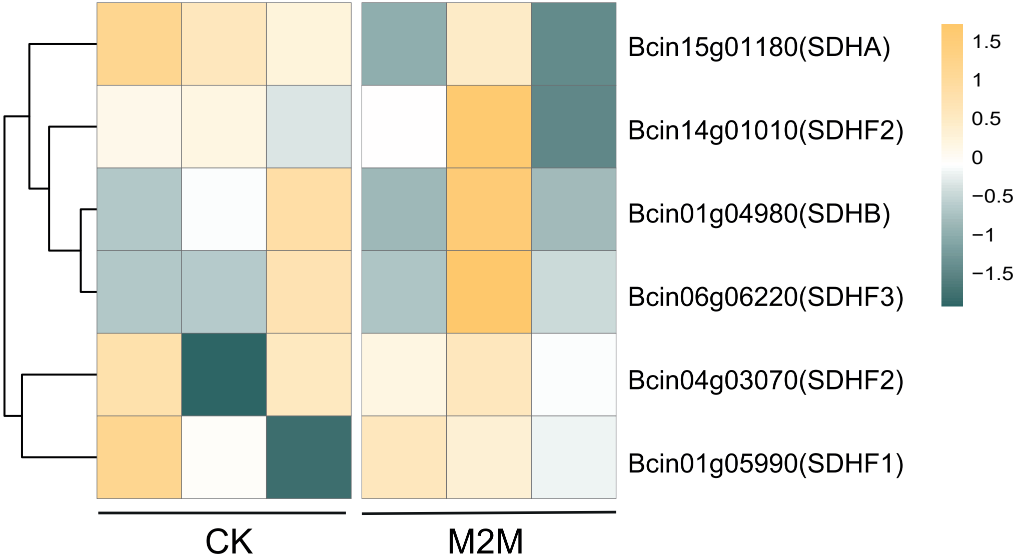
Bcin15g01180(SDHA)
Bcin14g01010(SDHF2)
Bcin01g04980(SDHB)
Bcin06g06220(SDHF3)
Bcin04g03070(SDHF2)
Bcin01g05990(SDHF1)
1.5
1
0.5
0
−0.5
−1
−1.5
CK
M2M
