# Supplementary material for: Antifungal Activity of Methyl 2-Methylbutyrate Against Botrytis cinerea and the Induced Physiological, Transcriptional, and Metabolic Responses
Source: Int J Mol Sci. 2026 Sep 25;27(19):8589. doi: 10.3390/ijms27198589 (PMC13665467; doi:10.3390/ijms27198589)
Supplement: Supplementary file 1 [file ijms-27-08589-s001.zip › Supplementary Table S1.pdf]

Supplementary Table S1. Raw physiological parameters of *Botrytis cinerea* in control (CK) and M2M-treated (Y) groups.

Parameter	Unit	Replicate	CK	Y
G6PDH	nmol/min/g	1	39.37	56.78
		2	36.26	58.64
		3	39.99	60.51
		Mean ± SE	38.54 ± 0.94	58.64 ± 0.88
MDA	nmol/g	1	72.03	28.42
		2	68.80	28.75
		3	66.86	26.81
		Mean ± SE	69.23 ± 1.24	27.99 ± 0.50
SOD	U/g	1	54.95	109.96
		2	56.96	108.15
		3	55.45	95.56
		Mean ± SE	55.79 ± 0.49	104.56 ± 3.79
CAT	μmoL/min/g	1	520.66	636.78
		2	533.41	604.21
		3	523.50	669.34
		Mean ± SE	525.86 ± 3.13	636.78 ± 15.34
POD	△OD470 /min/g	1	104.00	164.00
		2	97.00	172.00

Parameter	Unit	Replicate	CK	Y
		3	101.00	157.00
		Mean ± SE	100.67 ± 1.67	164.33 ± 3.54
SDH	U/g	1	140.59	263.41
		2	138.98	266.64
		3	132.51	269.87
		Mean ± SE	137.36 ± 2.02	266.64 ± 1.33
LDH	nmol/min/g	1	218.77	99.10
		2	223.71	98.11
		3	222.72	86.24
		Mean ± SE	221.73 ± 1.22	94.48 ± 3.29
